# The Herbst Clinics at Niagara

**Published:** 1886-08

**Authors:** 


### THE HERBST CLINICS AT NIAGARA.

Dr. Wilhelm Herbst will be at Niagara and will give clinics during Monday, the day preceding the meeting of the American Dental Association, and Tuesday, the first day of the meeting. It will be impossible for him to remain longer, as he sails for home during the week. It is unnecessary for us to urge that operative dentists be present at that time, for they will all recognize its importance. Here is a peculiar system of operating proposed, and it is the duty of everyone to become acquainted with its merits or demerits, no matter what their predilections may be. The candid man examines before he condemns or approves, and an opportunity that should be accepted is now offered to compare American with German manipulative skill, and finally to determine, each for himself, whether it is worth while to experiment with the rotary system of filling teeth.

